# Audiogram Shape: Does It Have a Significant Prognostic Role in Idiopathic Sudden Sensorineural Hearing Loss Outcome?

**DOI:** 10.3390/jpm15080379

**Published:** 2025-08-15

**Authors:** Gabriella Cadoni, Alberta Rizzuti, Michela Sollazzo, Pasqualina Maria Picciotti, Jacopo Galli

**Affiliations:** 1 ENT Department, Fondazione Policlinico Universitario Agostino Gemelli-IRCCS, 00168 Rome, Italy; gabriella.cadoni@policlinicogemelli.it (G.C.); michela.sollazzo@guest.policlinicogemelli.it (M.S.); pasqualinamaria.picciotti@policlinicogemelli.it (P.M.P.); jacopo.galli@policlinicogemelli.it (J.G.); 2 Department of Head and Neck and Sensory Organs, Università Cattolica del Sacro Cuore, 00168 Rome, Italy

**Keywords:** idiopathic sudden sensorineural hearing loss, audiogram shape, audiological outcomes, inflammatory markers, neutrophil-to-lymphocyte ratio (NLR), high-density lipoprotein (HDL)

## Abstract

**Background/Objectives:** Sudden sensorineural hearing loss (SSNHL) represents a challenging clinical entity with variable prognosis. Audiometric curve configuration has been proposed as a predictor of recovery. This study aimed to evaluate the association between audiogram morphology at onset and hearing outcome in patients with idiopathic unilateral SSNHL treated with standardized therapy. **Methods:** We retrospectively analyzed 156 patients with idiopathic SSNHL. Hearing thresholds at key frequencies were measured at baseline and 4 weeks post-treatment. Patients were categorized into upsloping, flat, downsloping, or U-shaped audiogram subgroups. Recovery was classified into four levels. Comparisons were made across subgroups for audiometric and laboratory data using ANOVA and chi-square tests. **Results:** Baseline PTA values were comparable across audiogram subgroups (*p* = 0.12). Hearing recovery differed significantly according to audiogram configuration (chi-square, *p* < 0.001), with upsloping and U-shaped patterns showing the best outcomes. Flat and downsloping curves were associated with poorer recovery, lower HDL, and elevated NLR values. **Conclusions:** Audiogram configuration is a relevant prognostic marker in SSNHL. Patterns linked to adverse metabolic and inflammatory profiles may benefit from tailored treatment strategies in a personalized medicine framework.

## 1. Introduction

Sudden sensorineural hearing loss (SSNHL) is an otolaryngological disorder defined as a rapid onset of hearing loss of at least 30 dB across three or more consecutive frequencies occurring within a 72 h period. It is typically unilateral and occurs without a known cause.

The estimated annual incidence in the United States is approximately 5 to 20 cases per 100,000 people, although more recent data suggest that the actual incidence may be higher due to increased awareness and improved diagnostic capabilities [1,2,3,4,5].

The disorder most commonly affects individuals between the fourth and sixth decades of life, with no consistent sex predilection.

Approximately 3% to 10% of cases occur in patients under the age of 18 years, but the true incidence is unknown in this age group [6].

Sudden sensorineural hearing loss is typically unilateral, with bilateral forms being rare and usually associated with systemic diseases. Patients often present with additional symptoms such as tinnitus, aural fullness, or vertigo, though these are not required for diagnosis. The onset is usually abrupt and the diagnosis is primarily audiometric, showing a sensorineural hearing loss pattern in the affected ear. Diagnostic evaluation aims to exclude identifiable causes through clinical assessment, imaging, and laboratory tests. Current American clinical guidelines recommend a prompt and thorough evaluation to rule out retrocochlear or systemic causes. Pure-tone audiometry remains the cornerstone for diagnosis, while contrast-enhanced magnetic resonance imaging (MRI) is essential for excluding vestibular schwannoma and other retrocochlear pathologies [2]. Several hypotheses have been proposed to account for the pathogenesis of sudden sensorineural hearing loss (SSNHL) [1,2]. Although a range of potential etiologies including neoplastic, infectious, autoimmune, neurologic, and ototoxic factors have been implicated, approximately 90% of cases remain idiopathic. To date, none of these mechanisms have been conclusively demonstrated. Treatment is most commonly initiated with systemic corticosteroids, either oral or intravenous, and in some cases, intratympanic steroid injections are used as primary or salvage therapy. Antioxidants, vasodilators, antivirals, or anticoagulants are not routinely recommended, as clinical trials have not demonstrated clear benefits [2,3,4]. Hyperbaric oxygen therapy (HBOT) is considered an optional treatment modality for idiopathic SSNHL and may be offered in combination with corticosteroids within 2 weeks of onset as initial therapy or within 1 month of onset as salvage treatment in patients who do not respond to corticosteroids alone. Nutritional supplements and alternative therapies remain investigational and are not part of standard treatment recommendations [2,5].

Approximately 32% to 65% of patients may experience some degree of spontaneous recovery, particularly within the first two weeks after onset [2]. Long-term follow-up is recommended to monitor hearing recovery and to identify an underlying cause that is not evident at initial presentation.

In cases where no recovery is observed, counseling and evaluation for hearing devices (such as hearing aids or cochlear implants) should be initiated. One of the major challenges in the management of idiopathic sudden sensorineural hearing loss (ISSNHL) is predicting treatment outcomes in order to tailor personalized therapeutic strategies, especially for patients at risk of poor recovery. The wide range of etiological hypotheses and the individual variability in clinical presentation contribute to a broad spectrum of recovery rates, underlining the need for more sophisticated prognostic tools.

Systemic inflammation plays a crucial role in the pathogenesis and prognosis of idiopathic sudden sensorineural hearing loss (ISSNHL), with simple hematological indices emerging as potential biomarkers. Among these, the neutrophil-to-lymphocyte ratio (NLR) has been shown to correlate inversely with hearing recovery in ISSNHL patients—higher NLR values are associated with poorer outcomes. Similarly, the platelet-to-lymphocyte ratio (PLR), though less consistently significant, appears to reflect inflammatory status and vascular function, which may influence cochlear recovery.

Building on this evidence, the present study aimed to evaluate whether the audiometric configuration at onset serves as a prognostic factor for hearing recovery in patients with idiopathic sudden sensorineural hearing loss (ISSNHL). Additionally, we investigated the potential association between systemic inflammatory markers—specifically, the neutrophil-to-lymphocyte ratio (NLR) and the platelet-to-lymphocyte ratio (PLR)—and both audiometric profiles and treatment outcomes, with the goal of enhancing individualized prognostic stratification.

## 2. Materials and Methods

### 2.1. Study Design and Patient Selection 

A retrospective observational analysis was conducted on a cohort of 156 patients diagnosed with idiopathic sudden sensorineural hearing loss (ISSNHL) who were evaluated and treated between January 2023 and October 2024 at the Day Hospital of the Agostino Gemelli University Hospital, Rome, Italy. Data were collected and analyzed in anonymized form to protect patient confidentiality. According to the institutional policy of our center, formal approval by the ethics committee was not required for this type of retrospective study. All patients provided written informed consent for the use of anonymized clinical data for research purposes. Patients underwent a thorough diagnostic workup, which included otoscopic evaluation, pure-tone and speech audiometry, tympanometry, auditory brainstem response (ABR), otoacoustic emissions (OAEs), clinical vestibular testing, videonystagmography, video head impulse test (v-HIT), and magnetic resonance imaging (MRI) of the brain and inner ear with and without contrast. In addition, all patients completed laboratory testing including routine bloodwork, autoimmune screening, microbiological panels, and coagulation profile assessment. From the complete blood count, we derived the neutrophil-to-lymphocyte ratio (NLR) and platelet-to-lymphocyte ratio (PLR), calculated by dividing the absolute neutrophil and platelet counts by the lymphocyte count, respectively. These systemic inflammatory indices were then analyzed in relation to audiometric subgroups and hearing recovery outcomes. Inclusion criteria were as follows: age ≥ 18 years; diagnosis of idiopathic unilateral sudden sensorineural hearing loss (iSSNHL) confirmed by pure-tone audiometry; symptom onset within 7 days prior to admission; and full adherence to the treatment protocol along with completion of the scheduled audiometric follow-up.

Exclusion criteria included a history of previous SSNHL in the same ear; the presence of concurrent middle ear or inner ear disorders of known origin (acoustic trauma, infectious or inflammatory otologic disease, perilymphatic fistula, retrocochlear lesions confirmed by magnetic resonance imaging, prior exposure to ototoxic agents, barotrauma, congenital malformations of the auditory system, definite Ménière’s disease). Additional exclusion criteria were the presence of vertigo or clinical signs of vestibular decompensation at presentation and incomplete diagnostic workup or audiological follow-up. This analysis was designed to explore prognostic stratification based primarily on audiogram configuration, aiming to identify hearing outcome patterns across different audiometric shapes to investigate their potential contribution to refining personalized treatment decision making in patients with ISSNHL.

### 2.2. Treatment Protocol

All patients received a standardized consecutive 5-day systemic therapy consisting of intravenous methylprednisolone at a dosage of 1 mg/kg/day and mannitol 18% solution administered over 20 min. Supportive care included intravenous hydration and gastroprotective medications.

Patients who did not show significant improvement after systemic therapy were additionally treated with salvage intratympanic dexamethasone injections (4 mg/mL) through a puncture of the posteroinferior portion of the tympanic membrane with a 25-gauge spinal needle.

Salvage therapy was initiated within one week after completion of systemic steroid treatment and consisted of a single intratympanic injection administered weekly, for up to three consecutive weeks. None of the patients received hyperbaric oxygen therapy (HBOT).

### 2.3. Audiological Assessment and Outcome Measures

Air conduction pure-tone average (PTA) thresholds were calculated for each ear using frequencies of 0.25, 0.5, 1, 2, and 4 kHz at baseline and at 4 weeks post-treatment.

**Figure 1:**
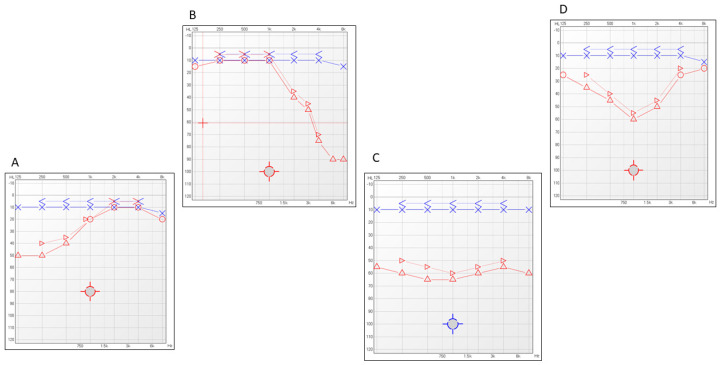
Representative examples of audiometric configurations observed at diagnosis. Four distinct patterns are illustrated: (**A**) Upsloping—characterized by greater hearing loss at lower frequencies with improvement at higher frequencies; (**B**) ownsloping—greater hearing loss at higher frequencies; (**C**) flat—similar hearing thresholds across all tested frequencies; (**D**) U-shaped thresholds at mid-frequencies compared with low and high frequencies.

These values were then used to categorize the degree of hearing loss at onset according to the following classification: mild (≥21 dB HL but <41 dB HL), moderate (≥41 dB HL but <71 dB HL), severe (≥71 dB HL but <91 dB HL), and profound (>91 dB HL). To classify the audiometric configurations, pure-tone audiograms were analyzed at the time of diagnosis. Based on the pattern of hearing loss across frequencies (250 Hz to 8000 Hz), the audiograms were categorized according to their configuration into four types: upsloping, characterized by greater hearing loss at low frequencies; downsloping, with greater loss at high frequencies; flat, indicating a uniform hearing loss across all tested frequencies; and U-shaped, defined by predominant hearing loss at mid-frequencies. Representative audiometric profiles by configuration at baseline are shown in Figure 1.

Audiological improvement was categorized according to the criteria described by Furuhashi et al. (2002) [7]. Complete recovery was defined as a post-treatment pure-tone average (PTA) between 0.25 and 4 kHz equal to or less than 25 dB HL. Marked improvement corresponded to a hearing gain greater than 30 dB HL, while slight improvement was defined as a gain between 10 and 30 dB HL. No recovery was assigned to cases with a hearing improvement of 10 dB HL or less.

### 2.4. Statistical Analysis

Statistical analysis was performed using IBM SPSS Statistics, version 27.0 (IBM Corp., Armonk, NY, USA). Descriptive statistics were used to summarize demographic and clinical characteristics.

One-way analysis of variance (ANOVA) was used to compare baseline pure-tone average (PTA) values and laboratory parameters across audiogram type subgroups. For laboratory parameters that showed statistically significant differences between groups, post hoc analysis was performed using Tukey’s Honestly Significant Difference (HSD) test. Particular attention was given to inflammatory indices such as neutrophil-to-lymphocyte ratio (NLR), platelet-to-lymphocyte ratio (PLR), and lipid profile components including high-density lipoprotein (HDL).

The chi-square test was applied to evaluate associations between audiogram configurations and patients’ clinical and demographic features as well as hearing recovery outcomes.

A *p*-value < 0.05 was considered statistically significant for all the analyses.

## 3. Results

A total of 156 patients with idiopathic sudden sensorineural hearing loss (iSSNHL) were analyzed and categorized according to audiometric curve type: upsloping (33.33%, *n* = 52), flat (25.64%, *n* = 40), downsloping (38.46%, *n* = 60), and U-shaped (2.56%, *n* = 4).

An overview of the main demographic and clinical features of the cohort is presented in Table 1.

The cohort included 98 men (62.8%) and 58 women (37.2%). Mean age varied across audiometric curve types: 41.25 ± 10.80 years in the upsloping group, 50.79 ± 15.17 years in the flat group, 44.71 ± 12.85 years in the downsloping group, and 44.54 ± 14.92 years in the U-shaped group. Laterality analysis showed that 60 patients (38.5%) had left-sided hearing loss, while 96 (61.5%) had right-sided involvement. Regarding smoking habits, 77 patients (49.4%) were smokers and 79 (50.6%) were non-smokers. The distribution of audiometric curve types differed significantly between genders. A large proportion of male patients presented with downsloping curves (*n* = 58), whereas female patients most frequently exhibited flat curves (*n* = 38) (χ^2^ = 87.39; df = 3; *p* < 0.001).

Baseline PTA values were broadly comparable across the different audiometric curve groups. Mean pre-treatment PTA ranged from 58.52 ± 5.89 dB HL in the downsloping group to 67.68 ± 3.87 dB HL in the U-shaped group. The mean PTA for upsloping and flat patterns was 62.3 ± 5.7 dB HL and 58.93 ± 5.6 dB HL, respectively. The upsloping group (*n* = 52) showed the most favorable outcomes, with 24 patients (46.2%) achieving complete recovery, 16 (30.8%) showing moderate recovery, 7 (13.5%) slight recovery, and 5 (9.6%) classified as non-responders. The U-shaped group (*n* = 4) exhibited excellent results, with 100% (4/4) of patients reaching complete recovery. In contrast, patients with flat audiograms (*n* = 40) had lower recovery rates: 5 (12.5%) achieved complete recovery, 10 (25.0%) moderate recovery, 7 (17.5%) slight recovery, and 18 (45.0%) showed no significant improvement. Similarly, in the downsloping group (*n* = 60), 10 patients (16.7%) reached complete recovery and 14 (23.3%) moderate recovery, while 12 (20.0%) had a slight recovery and 24 (40.0%) showed no benefit.

These findings are presented in the table below (Table 2), which summarizes hearing recovery outcomes across audiometric subgroups according to the classification system used by Furuhashi et al. (2002) [7].

**Figure 2:**
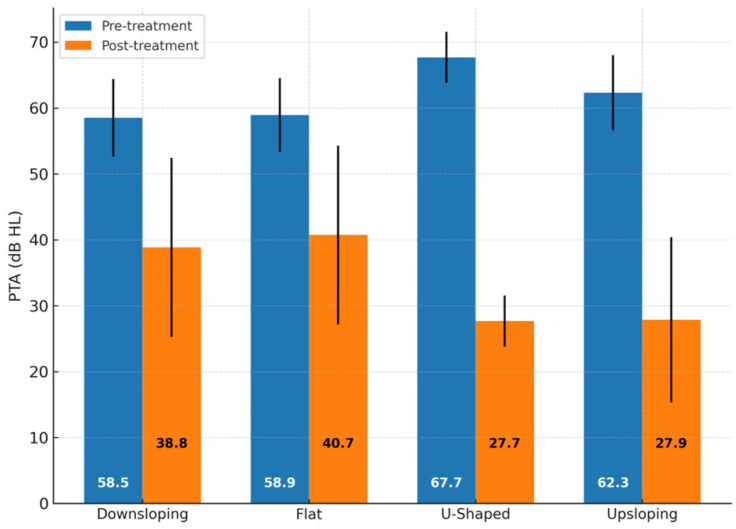
Comparison of pre- and post-treatment pure-tone average (PTA) values across audiogram shape subgroups. Mean PTA values before and after treatment are reported for downsloping, flat, U-shaped, and upsloping audiometric configurations. Baseline PTA values were broadly comparable across groups (mean range: 58.5–67.7 dB HL). Differences in PTA improvement were observed, with upsloping and U-shaped patterns showing greater post-treatment recovery compared with flat and downsloping patterns. No significant differences were detected in baseline PTA values across groups (one-way ANOVA, *p* = 0.12). Error bars indicate standard deviation. Abbreviations: PTA = pure-tone average; dB HL = decibels hearing level.

A summary of the mean PTA reduction (ΔPTA) calculated for each subgroup is shown in Figure 2, which reports baseline and post-treatment PTA values along with the corresponding ΔPTA for each audiometric subgroup.

The analysis of hematological parameters revealed significant differences among the audiometric subgroups. The neutrophil-to-lymphocyte ratio (NLR) varied significantly (*p* = 0.015), with the highest values observed in the downsloping group. Post hoc analysis (Tukey’s HSD) confirmed that NLR was significantly higher in the downsloping group compared with the upsloping and U-shaped groups (*p* < 0.001). The platelet-to-lymphocyte ratio (PLR) showed numerical differences across groups but did not reach statistical significance.

Among metabolic and lipid parameters, only high-density lipoprotein (HDL) levels differed significantly (ANOVA, F = 18.24, *p* < 0.001). Post hoc analysis revealed that HDL levels were significantly lower in the flat and downsloping groups than in the upsloping and U-shaped groups (*p* < 0.001). Notably, HDL values in the flat and downsloping groups were below standard reference ranges, supporting a potential link between impaired lipid metabolism and poorer auditory outcomes.

A comprehensive summary of laboratory testing across audiometric subgroups is provided in Table 3.

## 4. Discussion

This study explores the influence of audiometric curve configuration on hearing recovery outcomes in patients with idiopathic sudden sensorineural hearing loss (iSSNHL), suggesting that certain audiogram patterns may reflect distinct underlying pathophysiological mechanisms with prognostic implications. Hearing recovery varied across the audiometric subgroups. Patients with upsloping audiograms showed the most favorable outcomes, with 46.1% achieving complete recovery. This pattern may reflect a more reversible cochlear dysfunction, possibly involving the apical turn of the cochlea, which is thought to be less vulnerable to permanent ischemic damage. This interpretation is supported by previous findings from Psillas et al. [8] and Choo et al. [9], who demonstrated that patients with SSNHL limited to low frequencies had significantly better prognoses than those with high-frequency hearing loss [8,9,10,11]. Prior studies have indicated that the base of the cochlea may possess a higher susceptibility to injury compared with the apical region, potentially attributed to differences in vascular perfusion and metabolic requirements.

All audiometric subgroups presented average pre-treatment PTA values within the range of moderate hearing loss. The flat (58.9 dB HL) and downsloping (58.5 dB HL) groups showed nearly identical mean thresholds, while the upsloping group exhibited a slightly higher value (62.3 dB HL). The U-shaped subgroup showed the highest average PTA (67.7 dB HL), though it included only a very limited number of cases. These differences, however, remain within the same clinical severity category and do not appear to reflect a meaningful variation in hearing loss severity across audiogram types. All patients with U-shaped audiograms also achieved complete recovery. Although based on a small sample (*n* = 4), this observation suggests that mid-frequency involvement may be associated with a favorable prognosis. One possible explanation is that the regions of the cochlea responsible for mid-frequency hearing are less susceptible to irreversible injury or more responsive to corticosteroid treatment. Therefore, conclusions regarding this configuration should be interpreted with caution, as the small sample size (*n* = 4) limits the statistical strength and generalizability of the findings.

Patients with downsloping and flat audiograms demonstrated comparatively poorer recovery rates, with complete recovery observed in 16.7% and 12.5% of cases, respectively. These configurations may indicate more extensive or irreversible cochlear damage, such as outer hair cell loss or strial atrophy, particularly in the basal turn of the cochlea [12].

Many authors have explored the role of laboratory parameters—particularly markers of systemic inflammation—as both risk factors and potential prognostic indicators in SSNHL. However, the literature remains inconclusive, with conflicting results across studies. High white blood cell (WBC) levels may reflect immune-mediated cochlear ischemic processes that can lead to extensive inner ear tissue injury, as proposed by Kanzaki et al. [12]. Specifically, Seo et al. (2014) [3] and Kum et al. (2015) [13] found that patients with lower hearing improvement (less than 15 dB HL gain) had lower lymphocyte counts. In contrast, other authors such as Masuda et al. (2012), and Quaranta et al. (2015) did not find a significant association between lymphocyte levels and recovery outcomes, indicating that the role of lymphocytes in SSNHL prognosis remains uncertain and may be influenced by multiple coexisting factors [14,15,16,17].

Experimental models have proposed that the simultaneous activation of distinct NF-κB pathways may be required to trigger more severe forms of hearing loss. In this regard, a reduction in natural killer cell activity (NKCA), an acute-phase increase in neutrophil count, and elevated levels of IL-6 may act synergistically to induce NF-κB activation in the cochlea, promoting an enhanced inflammatory response and contributing to more extensive cochlear damage. These hypotheses offer a possible explanation for the association between inflammatory markers and limited auditory recovery [14,15,17].

In our study, patients with downsloping and flat audiograms were more likely to exhibit elevated systemic inflammatory markers such as the neutrophil-to-lymphocyte ratio (NLR). These findings suggest that underlying inflammatory or vascular mechanisms may contribute to the poorer auditory outcomes observed in these subgroups. In contrast, patients with upsloping and U-shaped audiograms generally exhibited lower levels of systemic inflammation, in line with their more favorable recovery profiles.

Lower values of HDLs, known for their anti-inflammatory and endothelial-protective properties, have been associated with an increased risk of microvascular impairment, which could contribute to cochlear hypoperfusion and reduced hearing recovery.

In our cohort, reduced HDL levels were predominantly observed in patients with flat and downsloping audiometric patterns, while those with upsloping and U-shaped configurations generally exhibited HDL values within the normal range. These observations support the idea that metabolic profiling, including HDL assessment and, potentially, fatty acid composition, could help identify patients with increased cardiovascular risk and predict less favorable auditory outcomes. The role of lipid dysmetabolism in SSNHL has been highlighted by Cadoni et al., who identified low serum levels of nervonic acid as an independent risk factor for the condition, supporting the hypothesis that altered fatty acid metabolism may contribute to cochlear vulnerability [18].

Numerous studies have established that the audiometric configuration, the time elapsed between symptom onset and initiation of therapy, and systemic inflammatory markers—such as neutrophil count—are independent prognostic variables influencing the outcome of SSNHL. These factors are considered critical in the early identification of patients more likely to benefit from treatment. In particular, both the type of the initial audiogram and the treatment delay have been repeatedly associated with differential recovery trajectories, making them reliable and accessible clinical indicators [1,16,19,20,21,22].

A recent study by Dong et al. (2024) developed a prognostic nomogram for SSNHL based on five clinical and audiometric parameters: audiogram configuration, presence of tinnitus, presence of vertigo, initial hearing level, and time from symptom onset to treatment initiation [17]. Their model demonstrates good predictive accuracy and reflects the growing interest in individualized risk stratification. Our findings align with this approach, particularly in supporting the prognostic relevance of audiogram shape.

In our study, we specifically focused on the prognostic value of audiometric configuration, analyzing its association with both post-treatment hearing outcomes and systemic laboratory parameters. Upsloping and U-shaped audiograms were associated with more favorable recovery profiles. These findings reinforce the clinical relevance of audiogram shape in prognostic stratification, particularly when interpreted alongside inflammatory markers such as neutrophil-to-lymphocyte ratio (NLR) and high-density lipoprotein (HDL).

The use of simple and accessible parameters such as audiometric pattern and basic blood markers may support a more personalized approach to the management of ISSNHL, allowing clinicians to tailor the intensity and timing of treatment based on an individual’s expected recovery profile. Further studies integrating additional known prognostic factors, such as treatment delay, are warranted to build more comprehensive and individualized prediction models.

Some limitations of this study should be acknowledged. The retrospective and single-center design and the relatively small number of patients in certain audiometric subgroups, such as the U-shaped pattern, may affect the statistical strength of subgroup comparisons. In addition, long-term outcomes were not evaluated. Nonetheless, the standardized treatment protocol and the inclusion of metabolic and inflammatory parameters represent strengths that support the relevance of the findings.

## 5. Conclusions

Audiometric curve configuration is a clinically relevant predictor of hearing recovery in SSNHL. Patterns associated with adverse metabolic and inflammatory profiles, particularly flat and downsloping audiograms, are linked to poorer outcomes and may warrant tailored therapeutic strategies. In this context, further studies are warranted to refine prognostic tools and guide personalized therapeutic approaches for patients with iSSNHL.

## Figures and Tables

**Table 1:** Demographic and clinical characteristics of the study cohort stratified by audiometric curve configuration. Data are reported as absolute numbers (with percentages, where applicable) or as mean ± standard deviation. Pure-tone average (PTA) values refer to the average hearing threshold calculated across the frequencies of 0.25, 0.5, 1, 2, and 4 kHz. Pre-treatment PTA indicates the degree of hearing loss at baseline, post-treatment PTA reflects the threshold after completion of therapy, and *ΔPTA* represents the mean improvement in hearing thresholds (in dB HL) following treatment.

Cohort	Upsloping	Flat	Downsloping	U-Shaped
**No. of patients**	52 (33.33)	40 (25.64)	60 (38.46)	4 (2.56)
**Gender**				
**Male**	35 (24.35)	2 (1.28)	58 (37.17)	3 (1.92)
**Female**	17 (41.47)	38 (24.35)	2 (7.6)	1 (0.64)
**Mean age**	41.25 ± 10.80	50.79 ± 15.17	44.71 ± 12.85	44.54 ± 14.92
**Side**				
**Left**	24 (58.53)	7 (17.50)	26 (43.33)	3
**Right**	28 (41.47)	33 (46.94)	34 (44.24)	1
**Smoking**				
**Yes**	16 (30.77)	14 (35)	31 (51.67)	1 (25)
**No**	36 (69.23)	26 (65)	29 (48.33)	3 (75)
**Tinnitus**				
**Yes**	28 (53.85)	15 (37.5)	33 (55)	1 (25)
**No**	24 (46.15)	25(625)	27 (45)	3(75)
**Treatment delay**	4.45 ± 2.17	5.17 ± 2.21	4.76 ± 2.74	5.93 ± 5.56
**Mean PTA 0.25**–**4 kHz**				
**Pre-treatment**	62.3 ± 5.7	58.93 ± 5.6	58.52 ± 5.89	67.68 ± 3.87
**Post-treatment**	27.87 ± 12.55	40.72 ± 13.54	38.83 ± 13.59	27.68 ± 3.87
**Delta PTA**	34.43 ± 14.28	18.21 ± 16.48	19.69 ± 15.87	40.00 ± 0.0

**Table 2:** Distribution of hearing recovery outcomes, based on the criteria of Furuhashi et al. [7], across the four audiometric curve subgroups. Values are expressed as absolute numbers and percentages. Patients with upsloping and U-shaped audiograms demonstrated higher rates of complete and moderate recovery, while flat and downsloping configurations were more frequently associated with no or slight recovery.

Audiogram Type	N	Complete Recovery (*n*, %)	Moderate Recovery (*n*, %)	Slight Recovery (*n*, %)	No Recovery (*n*, %)
**Upsloping**	52	24 (46.2%)	16 (30.8%)	7 (13.5%)	5 (9.6%)
**Flat**	40	5 (12.5%)	10 (25.0%)	7 (17.5%)	18 (45.0%)
**Downsloping**	60	10 (16.7%)	14 (23.3%)	12 (20.0%)	24 (40.0%)
**U-Shaped**	4	4 (100%)	0 (0%)	0 (0%)	0 (0%)

**Table 3:** Laboratory parameters across audiometric subgroups.

Parameter	Upsloping	Flat	Downsloping	U-Shaped	Comment
**Albumin** (**g/dL**)	4.3 ± 0.4	4.2 ± 0.3	4.1 ± 0.3	4.3 ± 0.4	ns
**Creatinine** (**mg/dL**)	0.88 ± 0.15	0.91 ± 0.16	0.95 ± 0.14	0.87 ± 0.13	ns
**BUN** (**mg/dL**)	14.6 ± 3.2	15.2 ± 3.4	16.0 ± 3.1	14.4 ± 2.9	ns
**Glucose** (**mg/dL**)	93 ± 12	95 ± 14	96 ± 15	92 ± 11	ns
**LDL** (**mg/dL**)	146 ± 26	138 ± 25	149 ± 28	135 ± 24	ns
**HDL** (**mg/dL**)	54 ± 10	38 ± 9	36 ± 8	52 ± 11	Lower in flat and downsloping
**AST** (**GOT**) (**U/L**)	23 ± 7	24 ± 6	25 ± 5	22 ± 6	ns
**ALT** (**GPT**) (**U/L**)	28 ± 9	29 ± 8	30 ± 7	26 ± 6	ns
**Triglycerides** (**mg/dL**)	145 ± 30	150 ± 33	155 ± 35	140 ± 28	ns
	**Upsloping**	**Flat**	**Downsloping**	**U-Shaped**	***p*-Value**
**Fibrinogen** (**mg/dL**)	297.49 ± 64.26	325.84 ± 77.33	295.18 ± 64.94	302.82 ± 76.04	ns
**aPTT**	27.46 ± 11.8	27.46 ± 13.57	27.81 ± 5.04	26.61 ± 4.97	ns
**PT**	98.62 ± 10.47	98.6 ± 12.54	97.09 ± 13.55	99.54 ± 10.6	ns
**Hemoglobin** (**g/dL**)	13.58 ± 1.43	13.59 ± 1.22	14.47 ± 1.36	14.01 ± 1.54	ns
**WBCs** (**10^3^/μL**)	9.1 ± 3.21	8.93 ± 2.62	9.58 ± 3.09	9.22 ± 3.01	ns
**Neutrophils** (**%**)	60.12 ± 12.91	75.2 ± 11.85	76.39 ± 10.84	67.09 ± 11.15	8.76 × 10^−1^
**Lymphocytes** (**%**)	28.5 ± 9.42	26.93 ± 9.71	23.18 ± 8.39	26.72 ± 9.23	ns
**NLR**	2.63 ± 1.63	2.98 ± 1.68	3.95 ± 1.83	3.12 ± 1.74	1.52 × 10^−7^
**PLR**	96.08 ± 25.25	122.11 ± 26.6	131.66 ± 29.07	106.62 ± 25.64	ns
**PC** (**10^3^/μL**)	246.48 ± 29.37	252.44 ± 29.95	266.57 ± 31.78	246.22 ± 28.25	ns
**Hematocrit** (**%**)	42.22 ± 2.64	40.07 ± 3.41	41.56 ± 3.27	42.96 ± 3.25	ns

The tables summarize the mean values (±standard deviation) of laboratory assessment according to audiometric curve configuration. One-way ANOVA was used to assess differences among groups; a *p*-value < 0.05 was considered statistically significant. Particular attention should be given to variations in NLR and HDL levels, which showed notable differences between the audiogram configurations.

## Data Availability

The data presented in this study are available on reasonable request from the corresponding author.
